# TMS Does Not Increase BOLD Activity at the Site of Stimulation: A Review of All Concurrent TMS-fMRI Studies

**DOI:** 10.1523/ENEURO.0163-22.2022

**Published:** 2022-08-03

**Authors:** Farshad Rafiei, Dobromir Rahnev

**Affiliations:** School of Psychology, Georgia Institute of Technology, Atlanta, GA 30313

**Keywords:** BOLD, concurrent TMS-fMRI, single-neuron recording

## Abstract

Transcranial magnetic stimulation (TMS) is widely used for understanding brain function in neurologically intact subjects and for the treatment of various disorders. However, the precise neurophysiological effects of TMS at the site of stimulation remain poorly understood. The local effects of TMS can be studied using concurrent TMS-functional magnetic resonance imaging (fMRI), a technique where TMS is delivered during fMRI scanning. However, although concurrent TMS-fMRI was developed over 20 years ago and dozens of studies have used this technique, there is still no consensus on whether TMS increases blood oxygen level-dependent (BOLD) activity at the site of stimulation. To address this question, here we review all previous concurrent TMS-fMRI studies that reported analyses of BOLD activity at the target location. We find evidence that TMS increases local BOLD activity when stimulating the primary motor (M1) and visual (V1) cortices but that these effects are likely driven by the downstream consequences of TMS (finger twitches and phosphenes). However, TMS does not appear to increase BOLD activity at the site of stimulation for areas outside of the M1 and V1 when conducted at rest. We examine the possible reasons for such lack of BOLD signal increase based on recent work in nonhuman animals. We argue that the current evidence points to TMS inducing periods of increased and decreased neuronal firing that mostly cancel each other out and therefore lead to no change in the overall BOLD signal.

## Significance Statement

Transcranial magnetic stimulation (TMS) is known to affect neuronal firing at the site of stimulation and can lead to downstream effects such as motor twitches. Consequently, it is widely assumed that TMS delivered inside a magnetic resonance imaging (MRI) scanner leads to an increase of blood oxygen level-dependent (BOLD) activity at the site of stimulation. Yet, the results in the literature are surprisingly mixed. Here, we comprehensively review all published concurrent TMS-functional MRI (fMRI) studies that report TMS effects on BOLD activity near site of stimulation. The review provides strong evidence for the surprising conclusion that TMS has no direct effects on BOLD activity at the site of stimulation. To understand the reason, we examine animal studies that reported how TMS affects neuronal firing at the site of stimulation.

## Introduction

Transcranial magnetic stimulation (TMS) is a noninvasive technique commonly used in both basic science research and clinical interventions (Kobayashi and Pascual-Leone, 2003; Ziemann, 2017; Chail et al., 2018). The neurophysiological effects of TMS at the site of stimulation have been explored by combining TMS with several neuroimaging techniques such as electroencephalography (Rossi, 2000; Paus et al., 2001; Strens et al., 2002; Oliviero et al., 2003; Thut et al., 2003), positron emission tomography (Fox et al., 1997; Paus et al., 1997, 1998; Siebner et al., 2001; Chouinard et al., 2003; Ferrarelli et al., 2004; Knoch et al., 2006), and single photon emission computed tomography (Okabe et al., 2003). A drawback for all of these methods, however, is their relatively low spatial resolution, which makes it difficult to resolve the activity induced specifically at the site of stimulation. Therefore, it has become increasingly popular to combine TMS with functional magnetic resonance imaging (fMRI) to examine how the stimulation affects the blood oxygen level-dependent (BOLD) signal immediately underneath the TMS coil (Bestmann et al., 2008a; Driver et al., 2009; Ruff et al., 2009).

The first study to employ concurrent TMS-fMRI was published in 1997 and focused on demonstrating the feasibility of the technique by examining the 3D intensity maps of the TMS-induced magnetic field inside a conventional MR scanner (Bohning et al., 1997). Since then, dozens of studies have used concurrent TMS-fMRI to reveal the local and distant effects of TMS during rest or task execution. The field has witnessed substantial improvements in the development of MRI-compatible TMS equipment including TMS coils, neuronavigation, stands, and even special MRI receiver coils designed for use in concurrent TMS-fMRI studies (De Weijer et al., 2014; Navarro de Lara et al., 2015; Wang et al., 2017; Goldstein et al., 2022). Such improvements have made it possible to investigate the BOLD activity at the precise site of stimulation with relatively high resolution and with negligible signal loss compared with standard fMRI.

However, despite all of these technical improvements and the availability of extensive research, the basic question of whether and under what conditions TMS affects the BOLD signal at the site of stimulation remains unresolved. Specifically, the field is yet to converge on which of the following two competing hypotheses is more likely:

- Hypothesis 1: The direct neural effects of TMS at the site of stimulation result in increased local BOLD activity.
- Hypothesis 2: The direct neural effects of TMS at the site of stimulation do not result in increased local BOLD activity.

The literature on concurrent TMS-fMRI is indeed seemingly divided with some studies finding increased local BOLD and others finding no BOLD increase at the site of stimulation. To date, there has been no comprehensive review of this literature specifically focused on the issue of local BOLD. The only paper that has examined this issue in more detail is a recent review by Bergmann et al. (2021), where the authors compiled all concurrent TMS-fMRI studies published until September, 2020. While Bergmann and colleagues do not specifically focus on the issue of local BOLD increases, they do provide a table regarding the details of all concurrent TMS-fMRI studies. That table includes a column to indicate whether local BOLD increase was observed in the study but does not separately examine the multiple conditions that are often present in a single study. Because of that, the table marks only one out of the 69 available studies as having found evidence for a lack of local BOLD increase. Without further examination of these studies, the classifications in this table can give the erroneous impression for the presence of overwhelming support for hypothesis 1 above. Indeed, Bergmann and colleagues also state that increased local BOLD is frequently not observed (thus acknowledging the viability of hypothesis 2) and provide an extensive discussion for the possible reasons. These considerations further highlighting the need for a comprehensive examination of whether the direct neural effects of TMS do or do not lead to increased local BOLD.

Here, we set to provide a comprehensive review of the published literature on concurrent TMS-fMRI with the goal of adjudicating between the two competing hypotheses above. First, we compiled all papers on concurrent TMS-fMRI published by December 31, 2021. To do so, we started with the 69 studies reported by Bergmann et al. (2021) and then, using equivalent search criteria, looked for additional studies published in 2020 and 2021, which resulted in five additional studies (Cobos Sánchez et al., 2020; Navarro de Lara et al., 2020; Jackson et al., 2021; Rafiei et al., 2021; Scrivener et al., 2021). Second, we excluded two sets of articles: (1) papers that primarily focused on methodological issues and only reported data from a single subject (Bestmann et al., 2003a, 2006; Peters et al., 2013; Navarro de Lara et al., 2015; Oh et al., 2019), and (2) papers that did not provide information that allows the determination of whether or not TMS led to an increase in local BOLD activation because relevant analyses were not reported (Ruff et al., 2006, 2008; Guller et al., 2012; Chen et al., 2013; Hanlon et al., 2016; Fonzo et al., 2017; Cobos Sánchez et al., 2020; Eshel et al., 2020; Hermiller et al., 2020; Navarro de Lara et al., 2020; Jackson et al., 2021; Oathes et al., 2021; Scrivener et al., 2021). Among the remaining studies, two pairs of studies shared the same underlying dataset (Leitão et al., 2013, 2015 and Shitara et al., 2011, 2013) and, therefore, we kept only the ones that explicitly mention results related to the presence or absence of activation at the site of stimulation (Shitara et al., 2011; Leitão et al., 2015). This process resulted in a total of 54 relevant articles. Of these, 22 articles delivered TMS outside of the primary motor (M1) or visual (V1) cortex at rest, 22 articles delivered TMS to M1 or V1 at rest, and 10 delivered TMS during a task.

We start by discussing factors critical to establishing the direct TMS effects on local BOLD activity (Factors Critical to Establishing the Direct TMS Effects on Local BOLD Activity). We then discuss each of the three groups of studies above separately in sections Studies That Targeted Areas Outside of M1/V1 during Rest, Studies That Targeted M1 or V1 during Rest, and Studies That Delivered TMS during a Task with the main focus falling on the articles that delivered TMS at rest outside of M1 and V1 (Studies That Targeted Areas Outside of M1/V1 during Rest). The review finds strong support for hypothesis 2 that the direct neural effects of TMS at the site of stimulation do not consistently result in increased local BOLD activity. We then explore how these findings relate to the available animal research regarding the effects of TMS on the firing rate of single neurons (The Effects of TMS on Neuronal Activity at the Site of Stimulation) and finish with a discussion of the possible reasons for why the direct neural effects of TMS may not lead to increased BOLD (Why Does Not TMS Have a Direct Effect on Local BOLD Activity?) and a brief conclusion.

## Factors Critical to Establishing the Direct TMS Effects on Local BOLD Activity

Establishing the direct BOLD effects of TMS at the site of stimulation requires the consideration of several factors. Here, we identify five factors that we believe are most critical to understanding the local effects of TMS (Table 1) and later examine each relevant study in sections Studies That Targeted Areas Outside of M1/V1 during Rest, Studies That Targeted M1 or V1 during Rest, and Studies That Delivered TMS during a Task in relation to these factors.

**Table 1:** Five critical factors

Factor	Why is this factor important?
Site of stimulation	Downstream effects of M1 or V1 stimulation may lead to different results for these sites compared with others
Task vs rest	TMS may have different effects based on whether a subject is at rest or engages in a task
Intensity and amount of stimulation	Higher intensities and more pulses may be more likely to affect the BOLD activity
Image artifacts	Spatial artifacts and signal dropout may occur near the TMS coil and mask genuine increases in BOLD activity at the site of stimulation
Precision of TMS localization and type of analyses	Imprecise localization of the site of stimulation increases the possibility for both false positives and false negatives; analyses on precisely localized ROIs are likely to be most informative

The table lists factors likely to be critical for establishing the direct effect of TMS at the site of stimulation and reasons for the importance of each factor. The first three factors relate to aspects of stimulation likely to affect the BOLD signal, whereas the last two factors are methodological.

### Site of stimulation

The local effects of TMS are likely not identical throughout the brain. Specifically, TMS to both M1 and V1 can lead to downstream consequences that a subject can directly experience (twitches in the contralateral hand or subjective visual experiences called “phosphenes”). Therefore, we consider the studies that targeted M1/V1 as a separate group (Studies That Targeted M1 or V1 during Rest).

### Rest versus task

The majority of concurrent TMS-fMRI studies are conducted at rest but an increasing proportion of studies are now conducted with an accompanying task to test how TMS modulates brain activity and connectivity. Because the effects of TMS on local BOLD activity may depend on whether the targeted region is already engaged in a task, we consider all studies conducted during a task as a separate group (Studies That Delivered TMS during a Task). This leaves all studies conducted during rest and targeting areas outside of M1 or V1 as the main focus here (Studies That Targeted Areas Outside of M1/V1 during Rest).

### The strength and amount of stimulation

Many aspects of TMS including intensity, frequency, as well as pattern and duration of stimulation may be important for the observed effects on the local BOLD signal (Aydin-Abidin et al., 2006; Fitzgerald et al., 2006; Krieg et al., 2015; Bergmann et al., 2016; Matheson et al., 2016). Therefore, we explicitly list these for all studies in sections Studies That Targeted Areas Outside of M1/V1 during Rest, Studies That Targeted M1 or V1 during Rest, and Studies That Delivered TMS during a Task.

### Image artifacts

The presence of the TMS coil inside the MRI scanner affects the homogeneity of the static magnetic field and can result in signal dropout near the site of stimulation (Baudewig et al., 2000; Bestmann et al., 2003a; Weiskopf et al., 2009; Bungert et al., 2012; Oh et al., 2019). This can be an important factor when examining the consequences of the magnetic stimulation underneath the coil. Since most studies in the literature do not explicitly measure or correct for TMS-related image artifacts, we discuss this issue at greater length in section Why Does Not TMS Have a Direct Effect on Local BOLD Activity?, where we examine all studies that did try to reduce image artifacts.

### Precision of TMS localization and type of analyses

Perhaps the most important factor for establishing the direct effects of TMS on local BOLD activity is the precision with which the site of stimulation has been localized (Romero et al., 2019). Precise localization allows the researcher to test only the site immediately underneath the coil with high power (e.g., without the need to correct for multiple comparisons). On the other hand, imprecise or nonexistent localization necessitates that a larger area is examined, which increases the likelihood of both false positives (e.g., an activation is found that is not at the true site of stimulation) and false negatives (e.g., the need to correct for multiple comparisons obscures a significant effect at the site of stimulation). Second-level analyses, which involve conducting across-subject analyses on normalized individual subjects’ brains, are likely to be particularly noisy because there is a large variability between the exact sites of stimulation for different subjects (Vink et al., 2018). Therefore, we treat the studies that precisely localized the site of stimulation for each subject and conducted region of interest (ROI) analyses as the gold standard for revealing the TMS effects on local BOLD activity. The type of localization and analysis is listed for all studies in sections Studies That Targeted Areas Outside of M1/V1 during Rest, Studies That Targeted M1 or V1 during Rest, and Studies That Delivered TMS during a Task.

## Studies That Targeted Areas Outside of M1/V1 during Rest

As highlighted above, arguably the studies most informative about the direct neural effects of TMS on local BOLD involve targeting areas outside of M1/V1 during rest. A total of 22 such papers have been published (Baudewig et al., 2001; Kemna and Gembris, 2003; Li et al., 2004a; Bestmann et al., 2005; Sack et al., 2007; Blankenburg et al., 2008; de Vries et al., 2009; X. Li et al., 2010; Hanlon et al., 2013; Leitão et al., 2013; De Weijer et al., 2014; Jung et al., 2016, 2020; Xu et al., 2016; Dowdle et al., 2018; Hawco et al., 2018; Kearney-Ramos et al., 2018; Vink et al., 2018; Peters et al., 2020; Webler et al., 2020; Rafiei et al., 2021) with one paper including two separate experiments (Rafiei et al., 2021). The authors reported significant BOLD increases at or near the site of stimulation for seven individual experiments, and no local change in BOLD for the remaining 16 experiments (Table 2). We examine the experiments from each group in more detail below.

**Table 2:** Studies delivering TMS outside of M1/V1 during rest

Study	Target(s)	Protocol(s)	Contrast(s)	*N*	Activation	Analyses
Li et al. (2004a)	L PFC	21 pulses over 21 s	100% rMT > rest	14	Yes	Second-level GLM
Bestmann et al. (2005)	L PMd	30 pulses over 10 s	(1) 110% rMT > 90% aMT (2) 110% rMT > rest	9	Yes	(1) ROI (anatomically defined) (2) Second-level GLM
Hanlon et al. (2013)	L DLPFC L MPFC	Single pulse	100% rMT > rest	17	Yes	ROI (anatomically defined)
Vink et al. (2018)	L DLPFC	Single pulse	115% rMT > 60% rMT	9	Yes	First-level GLMs
Hawco et al. (2018)	L DLPFC	Single pulse	100% rMT > 40% rMT	22	Yes	Second-level GLM
Peters et al. (2020)	R PMd	3 pulses over 66.67 ms	5% MSO below 100% rMT > rest	4	Yes	(1) ROI (functionally defined) (2) First-level GLMs
Webler et al. (2020)	L BA9	3 pulses over 100 ms	80%, 100% and 120% rMT > rest	18	Yes	ROI (anatomically defined)
Baudewig et al. (2001)	L PMC	10 pulses over 1 s	110% rMT > zero	6	No	ROI (functionally defined)
Kemna and Gembris (2003)	1) L PFC (2) L parietal	4 pulses over 1 s	150% rMT > zero	8	No	ROI (actual coil position)
Li et al. (2004b)	L PFC	21 pulses over 21 s	1) 120% rMT > rest (2) 100% rMT > rest (3) 120% rMT > 100%	8	No	Second-level GLM
Sack et al. (2007)	1) L SPL (2) R SPL	5 pulses over 300 ms	100% MSO > rest	8	No	Second-level GLM
Blankenburg et al. (2008)	R parietal	5 pulses over 500 ms	110% rMT > 50% rMT	5	No	Second-level GLM
De Vries et al. (2009)	L SPL	10 pulses over 10 s	115% rMT > rest	10	No	Second-level GLM
X. Li et al. (2010)	L PFC	5 pulses over 5 s	100% rMT > rest 120% rMT > rest	21	No	(1) ROI (anatomically defined) (2) Second-level GLM
Leitão et al. (2013)	R IPS	38 pulses over 20 s	(1) 66% MSO > 33% MSO (2) 66% MSO > rest	20	No	Second-level GLM
De Weijer (2014)	L dSMG	Single pulse	110% rMT > 70% rMT	3	No	(1) ROI (actual coil position) (2) First-level GLMs
Jung et al. (2016)	Vertex	12 pulses over 12 s	100% rMT > rest	32	No	Second-level GLM
Xu et al. (2016)	R pre-SMA	Single pulse	40% rMT > rest 80% rMT > rest 120% rMT > rest	17	No	(1) ROI (anatomically defined) (2) First-level GLMs
Dowdle et al. (2018)	L DLPFC	Single pulse	90–120% rMT > sham	20	No	(1) ROI (anatomically defined) (2) Second-level GLM
Kearney-Ramos et al. (2018)	R VMPFC	Single pulse	100% rMT > rest	49	No	(1) ROI (anatomically defined) (2) Second-level GLM
Jung et al. (2020)	Vertex	11 pulses over 11 s	100% rMT > rest	12	No	Second-level GLM
Rafiei et al. (2021), experiment 1	R PFC	(1) 20 pulses over 10 s (2) 10 pulses over 10 s	100% rMT > 50% rMT	5	No	(1) ROI (actual coil position) (2) First-level GLMs
Rafiei et al. (2021), experiment 2	L DLPFC	(1) 30 pulses over 1.2 s (2) 30 pulses over 2.4 s (3) 30 pulses over 3.6 s (4) 30 pulses over 6 s	100% rMT > rest	6	No	(1) ROI (actual coil position) (2) First-level GLMs

The table lists all 23 individual experiments using concurrent TMS-fMRI and stimulating areas outside M1 or V1 during rest. The first seven experiments reported significant activations in the vicinity of the TMS coil, whereas the remaining 16 reported no significant activations. aMT, active motor threshold; BA9, Brodmann area 9; DLPFC, dorsolateral prefrontal cortex; dSMG, dorsal supramarginal gyrus; GLM, general linear model; IPS, intraparietal sulcus; L, left; MPFC, medial prefrontal cortex; MSO, maximum stimulator output; PFC, prefrontal cortex; PMC, premotor cortex; PMd, dorsal premotor cortex; R, right; rMT, resting motor threshold; ROI, region of interest; SMA, supplementary motor area; SPL, superior parietal lobule; VMPFC, ventromedial prefrontal cortex.

### Studies reporting an increase in local BOLD activity

Seven papers reported local BOLD increase with TMS. The first three papers targeted the prefrontal cortex (PFC) and compared suprathreshold to subthreshold intensities using either first-level (Vink et al., 2018) or second-level analysis (Li et al., 2004a; Hawco et al., 2018). Each paper reported BOLD increases in many portions of the PFC including near the presumed location of stimulation. However, higher intensity TMS is louder and elicits more pronounced tactile sensations compared with low intensity TMS. This makes high intensity TMS feel more uncomfortable, which may induce emotional or cognitive changes, with the PFC known to be involved in both types of processes (Miller et al., 2002; Lorenz et al., 2003; Apkarian et al., 2005; Jobson et al., 2021). Further, given that these papers did not perform ROI-based analyses, it is impossible to determine whether the observed activations were indeed at the actual site of stimulation. Finally, in the case of one of the papers (Li et al., 2004a), the same authors used an identical TMS protocol in a different study and observed no significant activations (Li et al., 2004b). These considerations suggest that it is difficult to ascertain whether the activations reported in these three papers were because of direct neural effects of TMS at the actual site of stimulation.

The remaining four papers employed ROI analyses but none of them defined the ROIs based on the actual TMS coil location. Instead, three of them defined the ROI based on anatomic considerations (Bestmann et al., 2005; Hanlon et al., 2013; Webler et al., 2020), while the fourth used the functional activations from a different task (Peters et al., 2020). The first two papers targeted the dorsal premotor cortex (PMd; Bestmann et al., 2005; Peters et al., 2020). Bestmann et al. (2005) found that suprathreshold, but not subthreshold, stimulation evoked significant response in the PMd ROI, but, critically, did not report a statistical test on the comparison between the two types of stimulation. Peters et al. (2020) included four subjects in their study but primarily reported on the results from two of them (termed “high activators,” as opposed to the other two who were termed “low activators”), with again no statistical test reported either across subjects or for each of the four subjects separately. Thus, while both studies may contain statistically significant evidence for increased BOLD at the site of stimulation, this cannot be clearly determined from the reported information. Importantly, both studies also found widespread activations in motor-related areas of the brain, suggesting that TMS over PMd may result in generalized response in motor-related areas. It is therefore unclear whether the activations reported in PMd in these two papers were the result of the direct local neural effects of TMS, or a more generalized spreading of activation that reverberated throughout the motor network.

The remaining two papers that reported activation under the coil outside of M1/V1 during rest both targeted the PFC (Hanlon et al., 2013; Webler et al., 2020). Hanlon et al. (2013) targeted the dorsolateral PFC (DLPFC) and medial PFC (MPFC). Both areas were defined as ROIs for analyses using the large regions from the AAL atlas (Tzourio-Mazoyer et al., 2002). The authors found that TMS to either area produced increased activity in both ROIs, demonstrating that the activations observed in these analyses were not constrained to the site of stimulation. Nevertheless, the ROI corresponding to the targeted area showed greater activation, suggesting that local activity increase was greater. In the second study, Webler et al. (2020) used 80%, 100%, and 120% of rMT and examined activations in a large ROI that corresponds to Brodmann area 9 in a group of 11 schizophrenics and 8 controls. The authors reported a significant ROI activation for the 100% rMT in the subjects with schizophrenia (*p* = 0.0157), but no statistical tests were reported for the ROI activation for the other intensities or the control subjects.

### Studies reporting no increase in local BOLD activity

Fifteen papers including a total of 16 individual experiments reported no BOLD increases at the site of TMS (Baudewig et al., 2001; Kemna and Gembris, 2003; Li et al., 2004b; Sack et al., 2007; Blankenburg et al., 2008; de Vries et al., 2009; X. Li et al., 2010; Leitão et al., 2013; De Weijer et al., 2014; Jung et al., 2016, 2020; Xu et al., 2016; Dowdle et al., 2018; Kearney-Ramos et al., 2018; Rafiei et al., 2021). All papers used intensities equal to or higher than 100% of rMT. Seven of the papers only employed second-level GLMs (Li et al., 2004b; Sack et al., 2007; Blankenburg et al., 2008; de Vries et al., 2009; Leitão et al., 2013; Jung et al., 2016, 2020) and thus provide at best weak evidence against a direct neural effect of TMS on BOLD. Importantly, the remaining eight papers performed ROI-based analysis and thus provided much stronger evidence.

In the first five of the ROI-based papers, the ROIs were defined for each subject based on either the functional activations from a different task (Baudewig et al., 2001) or anatomic considerations (X. Li et al., 2010; Xu et al., 2016; Dowdle et al., 2018; Kearney-Ramos et al., 2018). The first study examined the hemodynamic response function (HRF) produced by TMS within the ROI and found that it does not differ from baseline (Baudewig et al., 2001). The other four studies found no effects of TMS both in the predefined ROIs and in second-level whole-brain analyses (X. Li et al., 2010; Xu et al., 2016; Dowdle et al., 2018; Kearney-Ramos et al., 2018). These findings serve as a counterweight to the positive findings above that were based on equivalent methods of using functionally or anatomically defined ROIs (Bestmann et al., 2005; Hanlon et al., 2013; Peters et al., 2020; Webler et al., 2020). However, as we have emphasized already, all of these studies should also be interpreted with caution given the lack of specificity in their ROI definitions.

The strongest evidence to date, therefore, comes from the remaining three papers that reported on four different experiments and used the gold-standard technique of precisely localizing the coil location for each subject (Kemna and Gembris, 2003; De Weijer et al., 2014; Rafiei et al., 2021). In the first such paper, Kemna and Gembris (2003) delivered TMS at 150% of rMT to the left prefrontal and parietal cortex at 4 Hz for 1 s. The authors extracted the time course of BOLD activity following TMS stimulation and found that it did not correlate with the canonical HRF in either the prefrontal or parietal cortex, although a significant correlation was observed in a separate condition where TMS was delivered to M1 and the activation in M1 was examined. In the second paper, De Weijer et al. (2014) found no BOLD activity difference between 110% of rMT and 70% of rMT stimulation of the supramarginal gyrus. Importantly, the study also measured the MRI signal quality at the exact spot of stimulation and did not find any signal dropout when compared with other brain regions. The final study was conducted by Rafiei and colleagues and consisted of two separate experiments both targeting DLPFC (Rafiei et al., 2021). In the first experiment, the authors included two conditions of suprathreshold stimulation (100% of rMT) and one condition of subthreshold stimulation (50% of rMT) where between 10 and 20 pulses were delivered over 10 s. In the second experiment, they used only 100% of rMT but delivered 30 pulses over 1.2 s (25 Hz), 2.4 s (12.5 Hz), 3.6 s (8.33 Hz), or 6 s (5 Hz). Despite the very large number of pulses in each burst, no condition in either experiment led to BOLD signal change at the site of stimulation as measured in ROIs of four different sizes. Importantly, these null results occurred although the TMS conditions could be decoded using multivoxel pattern analysis in the same ROIs in both experiments.

### Summary

Overall, the evidence to date strongly suggests that TMS delivered at rest outside of M1/V1 does not lead to increases in BOLD activity at the site of stimulation (thus supporting hypothesis 2 above). This conclusion was reached in all four individual experiments that used the gold-standard technique of precisely localizing the actual TMS coil position by using markers placed directly on the TMS coil (Kemna and Gembris, 2003; De Weijer et al., 2014; Rafiei et al., 2021). Further, the conclusion is also supported by the majority of the published literature (16/23 individual experiments). Finally, most of the remaining seven studies provided at best weak evidence for the notion that TMS leads to BOLD increases at the site of stimulation given that several of them did not feature ROI analyses at all, while others did not report direct statistical tests to establish local BOLD activations. Thus, the preponderance of evidence suggests that the direct neural effects of TMS delivered at rest outside of M1/V1 do not result in local BOLD increases.

## Studies That Targeted M1 or V1 during Rest

M1 and V1 are unique among typical TMS target locations in that stimulation to those areas produces effects that the participant has a subjective experience of (twitches for M1 TMS and phosphenes for V1 TMS) and are therefore examined separately here.

### Suprathreshold TMS induces a local BOLD increase

To date, 22 concurrent TMS-fMRI studies have targeted M1 or V1 with suprathreshold TMS intensities (i.e., intensities at or above 100% of resting motor threshold, rMT) during rest (Bohning et al., 1998, 1999, 2000a,b, 2003; Baudewig et al., 2001; Bestmann et al., 2003b, 2004; Kemna and Gembris, 2003; Denslow et al., 2005a,b; Hanakawa et al., 2009; Moisa et al., 2009, 2010; Caparelli et al., 2010; X. Li et al., 2010; Shitara et al., 2011; Yau et al., 2013; De Weijer et al., 2014; Jung et al., 2016, 2020; Navarro de Lara et al., 2017). All of these studies reported significant BOLD activations at or near the site of stimulation (Table 3). Twenty-one of these studies targeted M1, while only one of them targeted V1 (Caparelli et al., 2010).

**Table 3:** Studies targeting M1/V1

Study	Target	Protocol	Contrast(s)	*N*	Activation	Analyses
Bohning et al. (1998)	L M1	20 pulses over 24 s	110% rMT > rest	3	Yes	First-level GLMs
Bohning et al. (1999)	L M1	18 pulses over 17 s	110% rMT > rest	7	Yes	ROI (actual coil position)
Bohning et al., (2000b)	L M1	Single pulse	120% rMT > rest	5	Yes	ROI (actual coil position)
Bohning et al., (2000a)	L M1	21 pulses over 20 s	110% rMT > rest	7	Yes	Unknown
Baudewig et al. (2001)	L M1	10 pulses over 1 s	110% rMT > zero	6	Yes	ROI (functionally defined)
Bestmann et al. (2003b)	L M1	40 pulses over 10 s	110% rMT > zero	8	Yes	ROI (anatomically defined)
Kemna and Gembris (2003)	L M1	4 pulses over 1 s	150% rMT > zero	8	Yes	ROI (actual coil position)
Bohning et al. (2003)	L M1	1–24 pulses over 39 or 60 s	120% rMT > rest	4	Yes	ROI (actual coil position)
Bestmann et al. (2004)	L M1	30 pulses over 9.6 s	110% rMT > 90% aMT 110% rMT > rest	11	Yes	(1) ROI (anatomically defined) (2) Second-level GLM
Denslow et al., (2005b)	L M1	21 pulses over 21 s	110% rMT > rest	11	Yes	(1) ROI (anatomically defined) (2) Second-level GLM
Denslow et al., (2005a)	L M1	21 pulses over 21 s	110% rMT > rest	9	Yes	ROI (anatomically and functionally defined)
Hanakawa et al. (2009)	L M1	Single pulse	90% rMT > rest 100% rMT > rest 110% rMT > rest	16	Yes	(1) ROI (functionally defined) (2) Second-level GLM
Moisa et al. (2009)	L M1	10 pulses over 2 s	110% rMT > finger tapping cued by 50% rMT TMS	5	Yes	(1) ROI (anatomically defined) (2) Second-level GLM
Moisa et al. (2010)	L M1	16, 48, or 96 pulses over 24 s	100% rMT > VM-TMS 110% rMT > VM-TMS 120% rMT > VM-TMS	10	Yes	(1) ROI (anatomically defined) (2) Second-level GLM
Moisa et al. (2010)	L M1	48 pulses over 24 s	100% rMT > VM-TMS	10	Yes	(1) ROI (anatomically defined) (2) Second-level GLM
X. Li et al. (2010)	L M1	5 pulses over 5 s	100% rMT > rest 120% rMT > rest	25	Yes	(1) ROI (anatomically defined) (2) Second-level GLM
Caparelli et al. (2010)	L V1	8 pulses over 28 s	100% PT > rest	12	Yes	Second-level GLM
Shitara et al. (2011)	L M1	Single pulse	120% rMT > rest	36	Yes	(1) ROI (anatomically defined) (2) Second-level GLM
De Weijer (2014)	L M1	Single pulse	110% rMT > 70% rMT	4	Yes	(1) ROI (actual coil position) (2) First-level GLMs
Jung et al. (2016)	L M1	12 pulses over 12 s	100% rMT > vertex TMS	32	Yes	Second-level GLM
Navarro de Lara et al. (2017)	L M1	10 pulses over 10 s	100% aMT > rest 110% aMT > rest	7	Yes	(1) ROI (anatomically and functionally defined) (2) Second-level GLM
Jung et al. (2020)	L M1	11 pulses over 11 s	100% rMT > rest	12	Yes	Second-level GLM
Bohning et al. (1999)	L M1	18 pulses over 17 s	80% rMT > rest	7	No	ROI (actual coil position)
Baudewig et al. (2001)	L M1	10 pulses over 1 s	90% rMT > zero	6	No	ROI (functionally defined)
Bestmann et al. (2003b)	L M1	40 pulses over 10 s	110% aMT > zero 90% aMT > zero	8	No	ROI (anatomically defined)
Bestmann et al. (2004)	L M1	30 pulses over 9.6 s	90% aMT > rest	11	No	(1) ROI (anatomically defined) (2) Second-level GLM
Yau et al. (2013)	L M1	10 pulses over 10 s	90% rMT > finger tapping cued by 50% rMT TMS	8	No	(1) ROI (actual coil position) (2) Second-level GLM
Navarro de Lara et al. (2017)	L M1	10 pulses over 10 s	80% aMT > rest 90% aMT > rest	7	No	(1) ROI (anatomically and functionally defined) (2) Second-level GLM

The table first lists all 22 studies using suprathreshold stimulation first in order of publication. All six studies that reported subthreshold contrasts (reported in the second part of the table) also appear in the first part of the table. aMT, active motor threshold; GLM, general linear model; L, left; PT, phosphene threshold; rMT, resting motor threshold; ROI, region of interest.

Of the studies that found significant activations in M1, six used the gold-standard localization technique of defining an ROI at the precise site of stimulation using markers placed directly on the TMS coil (Bohning et al., 1999, 2000a, 2003; Kemna and Gembris, 2003; Yau et al., 2013; De Weijer et al., 2014), 11 defined ROIs based on either anatomic considerations or functional activations from a related task (Baudewig et al., 2001; Bestmann et al., 2003b, 2004; Denslow et al., 2005a,b; Hanakawa et al., 2009; Moisa et al., 2009, 2010; X. Li et al., 2010; Shitara et al., 2011; Navarro de Lara et al., 2017), four used first-level or second-level analyses (Bohning et al., 1998; Caparelli et al., 2010; Jung et al., 2016, 2020), and for one study the analysis type is unknown (Bohning et al., 2000a). Altogether, these studies paint an extremely consistent picture where TMS to M1 robustly increases BOLD activity at the site of stimulation.

A single concurrent TMS-fMRI study has targeted V1 (Caparelli et al., 2010). The authors stimulated at 100% of the phosphene threshold and found a significant BOLD increase in the visual cortex for subjects who experienced phosphenes but not for subjects who did not experience phosphenes. However, these results are based on second-level analyses and come from a single study, so they should be interpreted with caution.

### Subthreshold TMS does not increase the local BOLD signal

Six of the studies above included control conditions with subthreshold TMS intensities (i.e., intensities below 100% of rMT). All six of the studies found that subthreshold stimulation did not affect the overall BOLD activity at the site of stimulation (Bohning et al., 1999; Baudewig et al., 2001; Bestmann et al., 2003b, 2004; Yau et al., 2013; Navarro de Lara et al., 2017) even for intensities of up to 90% of rMT. In all of these cases, the null results were found in the same ROIs where a significant BOLD increase was found for suprathreshold stimulation. These results suggest that subthreshold TMS does not induce BOLD signal increases at the site of stimulation in M1.

### Summary

Overall, the studies that targeted M1 and V1 paint a very consistent picture: suprathreshold TMS intensities invariably lead to increases in local BOLD activity, whereas subthreshold intensities invariably lead to no significant local BOLD increases. Many researchers have speculated that this pattern of results emerges because the observed activations are caused by afferent feedback from contralateral muscle responses (Baudewig et al., 2001; Kemna and Gembris, 2003; Li et al., 2004b; X. Li et al., 2010; Bestmann et al., 2008a; Bestmann and Feredoes, 2013). The most direct evidence for this hypothesis comes from a study that compared TMS to M1 with a condition where electrical stimulation was applied to the right median nerve at the wrist (Shitara et al., 2011). The authors found that electrical activation at the wrist produced a very similar pattern of motor cortex (including M1) activations, thus confirming that many of the observed activations after M1 stimulation can be explained as being the result of feedback from muscle twitches. Similarly, as suggested by Caparelli and colleagues, BOLD increases in the primary visual cortex may be the result of higher-order areas that process the conscious experience of phosphenes sending feedback signals (Caparelli et al., 2010). Thus, although the possibility of direct neural effects cannot be completely excluded, it appears likely that in studies of M1 and V1, suprathreshold stimulation leads to local BOLD increases primarily because of its downstream consequences.

## Studies That Delivered TMS during a Task

TMS may be expected to have different effects on BOLD depending on whether the targeted brain region is engaged in a task or not. Specifically, it is unclear whether the conclusions obtained from studies conducted during rest should generalize to studies where the targeted brain area is already engaged in a task and thus may have elevated BOLD at the time of stimulation. To date, 10 papers reporting on 11 experiments have employed concurrent TMS-fMRI during a task and reported either presence or absence of local BOLD activation (Nahas et al., 2001; Sack et al., 2007; Bestmann et al., 2008c, 2010; Feredoes et al., 2011; Heinen et al., 2011, 2014; Ricci et al., 2012; Mason et al., 2014; Leitão et al., 2015, 2017). Of these, five experiments resulted in TMS-induced BOLD activity at the site of stimulation, while six experiments led to no such activity (Table 4).

**Table 4:** Studies conducted during a task

Study	Target	Task	Protocol	Contrast(s)	*N*	Activation	Analyses
Nahas et al. (2001)	L DLPFC	Tone discrimination	21 pulses over 21 s	120% rMT > rest 120% rMT > 80% rMT	5	Yes	Second-level GLM
Bestmann et al., (2008c)	L PMd	Grip	5 pulses over 455 ms	110% rMT > 70% aMT	12	Yes	(1) ROI (using coordinates from a previous study) (2) Second-level GLM
Feredoes et al. (2011)	R DLPFC	Working memory	3 pulses over 270 ms	110% rMT > 40% aMT	16	Yes	(1) ROI (using coordinates of the intended target)
Heinen et al. (2014)	R FEF	Visual attention task	3 pulses over 270 ms	110% rMT > 40% aMT	16	Yes	(1) ROI (using coordinates of the intended target)
Leitão et al. (2017)	R IPS	Spatial attention	4 pulses over 400 ms	69% MSO > sham TMS	8	Yes	Second-level GLM
Sack et al. (2007)	L SPL	Visuospatial judgment	5 pulses over 300 ms	100% MSO > task, no TMS	8	No	Second-level GLM
Bestmann et al. (2010)	Contralesional PMd	Grip	5 pulses over 455 ms	110% rMT > 70% aMT	12	No	Second-level GLM
Heinen et al. (2011)	R angular gyrus	Visuospatial attention	3 pulses over 270 ms	120% rMT > 40% rMT	5	No	(1) ROI (using coordinates of the intended target) (2) Second-level GLM
Ricci et al. (2012)	R PPC	Line bisection task	Single pulse	115% rMT > no TMS	3	No	First-level GLMs
Mason et al. (2014)	Wernicke’s area	Sentence comprehension task	300 pulses over 300 s	110% rMT > no TMS	26	No	Second-level GLM
Leitão et al. (2017)	R Occ	Spatial attention	4 pulses over 400 ms	69% MSO > sham TMS	8	No	Second-level GLM

The table lists all 11 experiments using concurrent TMS-fMRI and stimulating areas outside M1 or V1 during a task. The first five experiments reported significant activations in the vicinity of the TMS coil, whereas the remaining six reported no significant activations. aMT, active motor threshold; DLPFC, dorsolateral prefrontal cortex; FEF, frontal eye field; GLM, general linear model; IPS, intraparietal sulcus; L, left; MSO, maximum stimulator output; Occ, occipital cortex; PMd, dorsal premotor cortex; PPC, posterior parietal cortex; R, right; rMT, resting motor threshold; ROI, region of interest; SPL, superior parietal lobule.

**Table 5:** TMS studies performed in animals

Study	Species	Target	Protocol	Anesthetized?	*N*	A combination of increased and decreased firing rate?
Moliadze et al. (2003)	Cat	Primary visual cortex (area 17)	Single pulse	Yes	7	Yes (↑ ↓)
Allen et al. (2007)	Cat	Visual cortex	TMS bursts (1–4 s, 1–8 Hz)	Yes	8	No (↑)
Pasley et al. (2009)	Cat	Visual cortex	TMS bursts (1–4 s, 1–8 Hz)	Yes	2	No (↑)
Kozyrev et al. (2014)	Cat	Visual cortex	Single pulse, rTMS (10 Hz)	Yes	15	Yes (↑ ↓ ↑)
Mueller et al. (2014)	Monkey	FEF	Single pulse	No	2	Yes (neuron-dependent)
B. Li et al. (2017)	Rat	CFA	Single pulse	Yes	17	Yes (↑ ↓ ↑)
Romero et al. (2019)	Monkey	Parietal cortex	Single pulse	No	2	Yes (neuron-dependent)

The table lists all seven studies reporting TMS effects on neuronal activity. Five studies reported a combination of increasing and decreasing single neuron activity after stimulation. Two studies observed only increase in neuronal activity but the effects disappear after a few trials of single pulse stimulation. Arrows in the last column represent average increases and decreases in activity across the recorded neurons. FEF, frontal eye field; CFA, caudal forelimb area (rodent’s equivalent to the forelimb area of primate M1).

### Studies reporting an increase in BOLD activity

Of the five studies that found increased local BOLD activity, two used second-levels analyses and three used ROI-based analyses. The first two studies targeted either left DLPFC or right intraparietal sulcus (IPS) and reported significant activations near the presumed location of the TMS coil (Nahas et al., 2001; Leitão et al., 2017). The remaining three studies used predefined ROIs, though none of these studies used the gold-standard technique of defining the ROIs based on the actual coil position. The first study targeted PMd during a grip task or no grip rest (Bestmann et al., 2008c). The second study stimulated DLPFC time-locked to a working memory task (Feredoes et al., 2011). Finally, the third study targeted the frontal eye field (FEF) during a time-locked attention task (Heinen et al., 2014). In all three studies, suprathreshold TMS increased BOLD activity in the ROI compared with subthreshold TMS.

### Studies reporting no increase in BOLD activity

As a counterweight to the five studies that found TMS-induced increases of activity at the site of stimulation during tasks, six studies failed to find such increases. Of these, four studies employed various attention tasks and delivered TMS to attention-related brain areas such as the superior parietal lobule (Sack et al., 2007), angular gyrus (Heinen et al., 2011), posterior parietal cortex (Ricci et al., 2012), and occipital cortex (Leitão et al., 2017) in neurologically intact subjects (note that the last study reported significant BOLD activation in a separate condition that targeted IPS). Another study targeted Wernicke’s area during a sentence comprehension task again in normal subjects (Mason et al., 2014). The remaining study employed a motor task and stimulated PMd in a group of stroke patients (Bestmann et al., 2010). Four studies employed only second-level analyses (Sack et al., 2007; Bestmann et al., 2010; Mason et al., 2014; Leitão et al., 2017), one employed first-level analyses (Ricci et al., 2012), and only one employed ROI analyses with the ROIs defined based on the targeted coordinates (Heinen et al., 2011). Unfortunately, the lack of precise localization in the majority of these studies makes it hard to draw firm conclusions from them regarding the direct neural effects of TMS on the BOLD activity at the site of stimulation.

### Summary

Thus far, the concurrent TMS-fMRI studies that delivered TMS during a task paint an inconsistent picture of whether TMS leads to local BOLD increases. Five individual experiments found such increases, whereas six did not. Critically, none of the studies used the gold-standard technique of localizing the site of stimulation for each subject based on the actual coil location, and seven of the studies did not report ROI-based analyses at all. Thus, the heterogeneity in results and lack of precise localization make it difficult to draw strong conclusions about either the presence or absence of TMS-induced effects at the site of stimulation during tasks.

## The Effects of TMS on Neuronal Activity at the Site of Stimulation

Our review of the literature suggests that TMS appears not to have a consistent direct effect on the BOLD signal at the site of stimulation during rest. To understand the possible reasons for this, here we examine the TMS studies performed in nonhuman animals (hence referred to as “animals”) and discuss how what is known about the neuronal activity at the site of stimulation relates to the observed BOLD results.

To date, a total of seven published studies delivered TMS to animals and recorded single neuron firing at the site of stimulation (Moliadze et al., 2003; Allen et al., 2007; Pasley et al., 2009; Kozyrev et al., 2014; Mueller et al., 2014; B. Li et al., 2017; Romero et al., 2019). The studies varied in many aspects including species (cats or monkeys), TMS protocol (single pulses or trains), state of the animals (anesthetized or awake), and location of stimulation (visual cortex, FEF, parietal cortex, or motor cortex) (Table 5). More importantly, their findings are heterogeneous with no two studies from different labs finding similar effects of TMS. Nevertheless, one pattern of results appears in the majority of these studies: TMS typically produces a combination of both increases and decreases in single neuron activity.

Three studies found that TMS induces periods of increased and decreased firing. The first study delivered single TMS pulses to the primary visual cortex of anesthetized cats and found early activity increase (up to 500 ms after TMS) and a weaker but long-lasting activity decrease (up to 5–6 s after TMS; Moliadze et al., 2003). Another study delivered single TMS pulses to the caudal forelimb area (equivalent to primate motor cortex) of anesthetized rats and observed a very early activity increase (up to 50 ms after TMS), followed by a period of suppression (up to ∼200 ms after TMS), and another period of excitation (up to 300 ms after TMS; B. Li et al., 2017). Finally, a third study delivered single TMS pulses to the visual cortex of anesthetized cats and found a brief increase (< 20 ms after TMS) followed by a period of decreased activity (up to 400 ms after TMS), which then turned into increased activity again (lasting at least to 800 ms after TMS; Kozyrev et al., 2014). Critically, all three studies report results where the increases and decreases in single neuron activity seem to mostly cancel each other out.

Two recent studies delivered single-pulse TMS to FEF or the parietal cortex in awake monkeys and showed substantial differences in the response across neurons (Mueller et al., 2014; Romero et al., 2019). The studies uncovered neurons that only showed increases in firing, neurons that only showed decreases, and neurons that showed periods of both increases and decreases. These results are again consistent with the notion that TMS induces a mixture of increased and decreased neuronal activity.

Finally, two other studies delivered TMS to the visual cortex of anesthetized cats (Allen et al., 2007; Pasley et al., 2009). They found increased single neuron activity that lasted for a full minute (similar results were obtained using indirect measures of net activity including local field potentials, tissue oxygen, and total hemoglobin), but this effect diminished rapidly over the course of the experiment with some neurons showing no overall change in firing already by the fifth trial (Pasley et al., 2009). It should be noted that fMRI studies typically deliver hundreds of TMS trials, and therefore the BOLD effects in these studies primarily reflect the steady-state TMS influence on neural firing achieved after several dozen trials. Finally, both of these studies were conducted in anesthetized animals, leaving open the question of whether the same neuronal effects would be observed in the awake brain.

In summary, the animal literature on TMS consistently finds that TMS produces complex effects on neuronal firing with most studies reporting a combination of both increases and decreases of single neuron activity. None of the studies to date establish whether TMS changes the total amount of firing over the first few seconds after TMS (i.e., the period relevant for the observed BOLD response) at the site of stimulation. Yet, at least some studies suggest that the TMS-induced a pattern of activity increases and decreases that may be balanced such that there is little to no overall increase in firing during the first 1–2 s after TMS.

## Why Does Not TMS Have a Direct Effect on Local BOLD Activity?

The current review strongly suggests that TMS does not have a direct effect on local BOLD activity. Many potential reasons for a lack of local BOLD activity have been discussed in the literature (Bergmann et al., 2021). Here, we compile and critically examine the previously discussed explanations.

### TMS stimulation is too weak or short

One potential reason for a lack of TMS effect on local BOLD could be that the applied stimulation was too weak or short (Reithler et al., 2011; Bergmann et al., 2021). Indeed, low-intensity TMS would logically result in smaller effects than high-intensity TMS and a single TMS pulse could be expected to have a smaller effect than a train of TMS pulses. Nevertheless, it is difficult to see how these considerations alone could explain the results in the literature. For example, out of the seven experiments that found local BOLD activity increase outside of M1/V1 (see Table 2), four employed trains of pulses (57%) and only two employed intensities above 100% of rMT (29%). On the other hand, out of the 16 experiments that found no local BOLD activity increase outside of M1/V1 (see Table 2), 12 employed trains of pulses (75%) and 11 employed intensities above 100% of rMT (69%). In other words, the studies that failed to find local BOLD increases used more pulses and higher intensities. Thus, the difference between the two sets of studies does not appear to stem from the intensity and length of stimulation.

### Low signal-to-noise ratio (SNR) at the site of stimulation

Another possible reason for the lack of BOLD increase at the site of stimulation is that there may be image distortion or a signal drop underneath the TMS coil, which could mask genuine increases in the BOLD signal (Baudewig et al., 2000; Bestmann et al., 2003a, 2008a; Blankenburg et al., 2008; Bergmann et al., 2021). Several remedies have been employed to reduce the effect of the presence of the TMS coil on the static magnetic field in nearby areas. One commonly used method to reduce such artifacts is to make frequency-encoding direction and slice orientation parallel to the TMS coil (Bestmann et al., 2003a; Moisa et al., 2009; Weiskopf et al., 2009; Bungert et al., 2012; Navarro de Lara et al., 2015, 2017). Other techniques intended to minimize the static artifacts and signal loss include using a relay-diode combination (Weiskopf et al., 2009), passive shimming (Bungert et al., 2012), customized radio frequency arrays (Navarro de Lara et al., 2015, 2017), and using B0 field maps or point spread function (Oh et al., 2019). Importantly, the papers above that used various methods for minimizing image artifacts were not more likely to find increased BOLD activations at the site of stimulation compared with other papers that did not specifically try to minimize image artifacts. Specifically, five studies that targeted areas outside M1 during rest and corrected for distortion effects during preprocessing did not find any activations at the site of stimulation (Kemna and Gembris, 2003; Leitão et al., 2013; De Weijer et al., 2014; Xu et al., 2016; Dowdle et al., 2018), while only a single study that targeted areas outside M1 during rest and corrected for image artifacts using a field map reported local BOLD activations in a second-level analysis (Hawco et al., 2018).

Another critical issue to consider is the improvement in TMS technology over the years. Early studies using Dantec technology (Bohning et al., 1997, 1999, 2000a, b) induced substantial dropout that likely substantially affected the SNR in the vicinity of the coil (Weiskopf et al., 2009). However, modern Magstim and especially MagVenture setups typically result in excellent signal underneath the TMS coil (De Weijer et al., 2014; Bergmann et al., 2021). Indeed, several studies have explicitly measured signal dropout using MagVenture equipment and have found it to be minimal (Moisa et al., 2009, 2010; Leitão et al., 2013). Further, the image distortions and dropout reduce with the distance from the coil. Given the typical scalp-to-cortex distance of 1–2 cm, the distortions in the brain are typically small (Bestmann et al., 2008b). Importantly, the signal drop even in early studies was not large enough to prevent the reliable detection of BOLD activations in M1 when that region was targeted (Bohning et al., 1997, 1998, 1999, 2000a,b; Moisa et al., 2009; De Weijer et al., 2014). Thus, the image artifacts caused by the presence of the TMS coil appear unlikely to fully explain the lack of local BOLD increases uncovered in the present review, especially for studies that correct for image artifacts or use modern technology that has been shown to result in minimal levels of distortion.

### Nonstandard HRF shape

A third possibility is that the BOLD response produced by TMS pulses does not follow the shape of the standard HRF (Bergmann et al., 2021). To test this possibility, several studies have performed finite impulse response analyses to explore the actual shape of the BOLD response independently of any assumptions (Bohning et al., 1999, 2000b; Baudewig et al., 2001; Bestmann et al., 2003b; Kemna and Gembris, 2003; Rafiei et al., 2021). These studies either found no significant BOLD increase at any time point (Bohning et al., 1999; Baudewig et al., 2001; Kemna and Gembris, 2003; Rafiei et al., 2021), or, for cases when a change was observed such as after M1 stimulation, the response was similar to the standard HRF shape (Bohning et al., 2000b; Baudewig et al., 2001; Bestmann et al., 2003b; Kemna and Gembris, 2003). Further, several studies that failed to find local TMS-related BOLD increases used long intervals of stimulation that obviate the need for precise HRF modeling (Li et al., 2004b; de Vries et al., 2009; X. Li et al., 2010; Leitão et al., 2013; Jung et al., 2016, 2020; Rafiei et al., 2021). Therefore, a nonstandard TMS-induced HRF shape is also unlikely to account for the lack of observed BOLD increase.

### Low number of subjects

A fourth possibility for the lack of BOLD increase at the site of stimulation is that most concurrent TMS-fMRI studies employ a low number of subjects and this results in low statistical power. Indeed, the median sample size among the 52 studies reviewed here is 8. Importantly, this concern does not apply to the studies that examined the effects of TMS on each individual, but they do affect the studies that performed group-level analyses. Nevertheless, it should be noted that M1 activation is robust even in studies with few number of subjects (Bohning et al., 1998, 2000a, 2003; Moisa et al., 2009; De Weijer et al., 2014), whereas even the top three studies with largest number of subjects (Mason et al., 2014; Jung et al., 2016; Kearney-Ramos et al., 2018) did not find significant activation when TMS was applied over areas outside of M1/V1. Thus, while the low number of subjects remains an important concern, this issue alone does not seem to explain the pattern of results in the literature.

### TMS may affect primarily the output of a given area and thus not lead to local BOLD activity

A fifth possibility for the lack of local BOLD increase is that TMS may mostly affect the output of the stimulated brain area (Baudewig et al., 2001; X. Li et al., 2010), whereas BOLD activity primarily reflects the input and intracortical processing of a given area rather than its spiking output (Logothetis, 2003; Logothetis and Wandell, 2004). However, TMS is thought to also affect intracortical processing including perisynaptic and postsynaptic activity, which should lead to increased BOLD at the site of stimulation (Kemna and Gembris, 2003; Di Lazzaro et al., 2008; Bergmann et al., 2021). Because relatively little is known about the exact type of neuronal activity elicited by TMS and its associated neurovascular coupling and oxygen utilization demands, this explanation for the lack of local BOLD increases is difficult to evaluate at present.

### TMS produces a combination of increases and decreases in neuronal activity that balance out

Finally, the lack of local BOLD increase may be because of TMS producing a pattern of increased and decreased single neuron activity that mostly balance each other out (Kemna and Gembris, 2003; Bergmann et al., 2021; Rafiei et al., 2021). This hypothesis is based on several animal studies that demonstrate that TMS induces a combination of increased and decreased neuronal activity (Moliadze et al., 2003; Allen et al., 2007; Pasley et al., 2009; Kozyrev et al., 2014; Mueller et al., 2014; B. Li et al., 2017; Romero et al., 2019). Notably, similar increases and decreases in neuronal activity have also been found in animals during electrical stimulation (Krnjević et al., 1964). Critically, the decreases in single neuron activity elicited by electrical stimulation appear to result from long-lasting γ-aminobutyric acid release (that likely does not affect BOLD) rather than continuous inhibitory neuronal activity (that may have caused BOLD increases; Krnjević et al., 1964). While the possibility of relatively precise balance of increases and decreases in neuronal activity is also speculative, we argue that this is currently the most likely explanation for the lack of BOLD increases associated with the direct neural effects of TMS.

## Conclusion

Here we reviewed all published concurrent TMS-fMRI studies that report the effects of TMS on BOLD activity at or near the site of stimulation, and attempted to adjudicate between the hypotheses that TMS does or does not have a direct neural effect on local BOLD activity. The review strongly suggests that TMS does not have a direct neural effect on BOLD activity at the site of stimulation. The strongest evidence comes from studies that delivered TMS outside of M1/V1 during rest: the great majority of them found no local BOLD changes. Studies targeting M1/V1 are also consistent with this interpretation since it appears that they only lead to BOLD increases in the presence of feedback related to hand twitches or phosphenes. Our results demonstrate that local BOLD activity increases should not be automatically expected when delivering TMS and cannot be used as a robust method of verifying the effectiveness of the TMS stimulation. We speculate that the most likely explanation for the lack of direct TMS-induced BOLD changes at the site of stimulation is that TMS induces a combination of increases and decreases in neuronal activity underneath the coil that balance each other out. This explanation suggests the presence of robust regulatory mechanisms that dynamically adjust the overall firing in an area in the presence of artificially induced firing. An exciting possibility is that such regulatory processes may be disrupted in disorders such as epilepsy and that TMS could provide a promising avenue for studying their mechanisms.

## Synthesis

Reviewing Editor: Alexander Soutschek, Ludwig-Maximilians-Universitat Munchen Decisions are customarily a result of the Reviewing Editor and the peer reviewers coming together and discussing their recommendations until a consensus is reached. When revisions are invited, a fact-based synthesis statement explaining their decision and outlining what is needed to prepare a revision will be listed below. The following reviewer(s) agreed to reveal their identity: Marius Moisa.

The authors provide a comprehensive review of the previous concurrent TMS-fMRI studies to address how TMS induces brain response at the site of stimulation. They reviewed relevant papers on concurrent TMS-fMRI, including delivered TMS outside of the primary motor (M1) or visual (V1) cortex, delivered TMS to M1 or V1, and TMS during a task. They have two hypotheses: 1) The direct neural effects of TMS at the site of stimulation result in increased local BOLD activity, and 2) The direct neural effects of TMS at the site of stimulation do not result in increased local BOLD activity. They found evidence that TMS increases local BOLD activity only when stimulating M1 or V1, but these effects are likely related to the finger twitches and phosphenes. However, they didn’t find increased local BOLD activity when stimulating outside of M1 and V1, and they argue that TMS inducing increased and decreased neuronal firing may cancel each other. In general, this paper is well written and covered all papers on concurrent TMS-fMRI. The reviewers agree that the work is important to the concurrent TMS-fMRI studies. However, there are some issues that need to be addressed before publication.

The authors identify four factors for establishing the direct effect of TMS at the site of stimulation (Table 1). However, given the findings related to image artifacts from previous TMS-fMRI papers (Bestmann et al., 2003, Weiskopf et al., 2009, Bungert et al., 2012, Oh et al., 2019), the authors should add one more factor - image artifacts caused by the TMS coil, especially underneath the TMS coil. B0 field maps from previous studies already demonstrated that there were significant signal dropouts near the TMS coil. Concurrent TMS-fMRI studies may not be able to find increased or decreased BOLD responses at the site of stimulation because of local signal loss in EPI data. Therefore, the authors should include any concurrent TMS-fMRI studies with detailed information on how those studies characterized the static spatial artifacts and signal loss, even though they briefly mentioned the signal drop in Section 7.

Minor concerns:

1) As a reader, it would be helpful if the authors provide a table describing Section 6 (i.e., animal studies). The table should include a similar format to other tables (e.g., species, TMS protocol, TMS location, findings, etc.).

2) The authors should carefully correct the reference format (e.g., Allen RA, Psaley BN, Doung T, Freeman RD (2007) Transcranial magnetic stimulation elicits coupled neural and hemodynamic consequences. Science (1979) 317:1918-1921.).

3) Line 90. Please mention how many studies have been added, i.e. published in 2020 and 2021.

4) Line 97. There seems to be a mismatch between the number of published TMS-fMRI and the studies discussed here. The authors state that they excluded 15 papers from the 69 studies published till 2019, i.e. discussed in by Bergmann and colleagues (Bergmann et al., 2021), plus the papers publishes in 2020 and 2021. However, the final included number of papers addressed in this review is 52. Can the authors explain this mismatch? Or even briefly discussed the findings of any paper that was not yet included in this review. Thank you!

5) Line 190: Can the authors motivate why “louder” pulses and “more pronounced tactile sensations” might elicit higher activation in the prefrontal cortex?

6) Line 459: Does any of two studies discuss the net effect of the stimulation on the local activity?

7) Line 474. Section 7. The authors should also discuss the fact that most of the TMS-fMRI studies tested a relatively low number of subjects, e.g. compared with the number of subjects employed in standard fMRI studies. For example, in the three studies published most recently, i.e. Jung et al. 2020 (12 subjects for each experimental group), Peters et al. 2020 (4 subjects), Rafiei et al. 2021 (5 subjects in Experiment 1 and 6 subjects in Experiment 2). It is true that this is not relevant for individual effects of the stimulation; however, this could be a potential issue when addressing group level effects of TMS at the stimulation site.

## Author Response

Dear Dr. Soutscheck,

Thank you for giving us this opportunity to revise our manuscript. We found the comments from the reviewers very helpful and constructive, and have now carefully addressed all of them. We provide a point-by-point response to all comments below.

Sincerely,

The Authors

Major concern

The authors identify four factors for establishing the direct effect of TMS at the site of stimulation (Table 1). However, given the findings related to image artifacts from previous TMS-fMRI papers (Bestmann et al., 2003, Weiskopf et al., 2009, Bungert et al., 2012, Oh et al., 2019), the authors should add one more factor - image artifacts caused by the TMS coil, especially underneath the TMS coil. B0 field maps from previous studies already demonstrated that there were significant signal dropouts near the TMS coil. Concurrent TMS-fMRI studies may not be able to find increased or decreased BOLD responses at the site of stimulation because of local signal loss in EPI data. Therefore, the authors should include any concurrent TMS-fMRI studies with detailed information on how those studies characterized the static spatial artifacts and signal loss, even though they briefly mentioned the signal drop in Section 7.

We thank the reviewers for alerting us with this concern. We also think that image artifact is an important factor and, therefore, included this factor in Table 1 of our manuscript. Inhomogeneity in static magnetic field was reported in several studies in the literature and several methods were suggested to minimize the effect. Because relatively few studies have addressed this issue, we felt that it is more appropriate for us to expand our discussion in Section 2 (where Table 1 is located) and Section 7 (where we discuss possible reasons for our findings). Therefore, we follow the request to include all concurrent TMS-fMRI studies that addressed the issues of static spatial artifacts and signal loss by including them in Section 7.

Overall, we feel that while the issue of image artifacts is an important factor to discuss and be aware of, it is unlikely to explain the pattern of results in the literature. This is for several reasons that we highlight, including the fact that (1) such artifacts are relatively small in modern setups especially for MagVenture equipment, (2) studies that attempted to correct for such artifacts usually found the same results as the rest of the literature, and (3) studies that targeted M1 invariably found local BOLD activations even in the case of the earliest studies that suffered from the largest image artifacts. If the reviewers feel that these arguments or conclusions are overstated, we would be happy to qualify them further as necessary. Below, we paste the updated Table 1, the corresponding brief part in Section 2 devoted to image artifacts, and the expanded part of Section 7 where we discuss these issues in much greater detail than before:

Page 7:

Table 1. Five critical factors. The table lists factors likely to be critical for establishing the direct effect of TMS at the site of stimulation and reasons for the importance of each factor. The first three factors relate to aspects of stimulation likely to affect the BOLD signal, whereas the last two factors are methodological.

Factor Why is this factor important?

Site of stimulation Downstream effects of M1 or V1 stimulation may lead to different results for these sites compared to others

Task vs. rest TMS may have different effects based on whether a subject is at rest or engages in a task

Intensity and amount of stimulation Higher intensities and more pulses may be more likely to affect the BOLD activity

Image artifacts Spatial artifacts and signal dropout may occur near the TMS coil and mask genuine increases in BOLD activity at the site of stimulation

Precision of TMS localization and type of analyses Imprecise localization of the site of stimulation increases the possibility for both false positives and false negatives; analyses on precisely localized ROIs are likely to be most informative

Page 8:

Image artifacts

The presence of the TMS coil inside the MRI scanner affects the homogeneity of the static magnetic field and can result in signal dropout near the site of stimulation (Baudewig et al., 2000; Bestmann et al., 2003a; Bungert et al., 2012; Oh et al., 2019; Weiskopf et al., 2009). This can be an important factor when examining the consequences of the magnetic stimulation underneath the coil. Since most studies in the literature do not explicitly measure or correct for TMS-related image artifacts, we discuss this issue at greater length in Section 7 where we examine all studies that did try to reduce image artifacts.

Page 28:

Low signal-to-noise ratio (SNR) at the site of stimulation

Another possible reason for the lack of BOLD increase at the site of stimulation is that there may be image distortion or a signal drop underneath the TMS coil, which could mask genuine increases in the BOLD signal (Baudewig et al., 2000; Bergmann et al., 2021; Bestmann et al., 2008a, 2003a; Blankenburg et al., 2008). Several remedies have been employed to reduce the effect of the presence of the TMS coil on the static magnetic field in nearby areas. One commonly used method to reduce such artifacts is to make frequency-encoding direction and slice orientation parallel to the TMS coil (Bestmann et al., 2003a; Bungert et al., 2012; Moisa et al., 2009; Navarro de Lara et al., 2017; Navarro De Lara et al., 2015; Weiskopf et al., 2009). Other techniques intended to minimize the static artifacts and signal loss include using a relay-diode combination (Weiskopf et al., 2009), passive shimming (Bungert et al., 2012), customized radiofrequency arrays (Navarro de Lara et al., 2017; Navarro De Lara et al., 2015), and using B0 field maps or point spread function (Oh et al., 2019). Importantly, the papers above that used various methods for minimizing image artifacts were not more likely to find increased BOLD activations at the site of stimulation compared to other papers that did not specifically try to minimize image artifacts. Specifically, five studies that targeted areas outside M1 during rest and corrected for distortion effects during preprocessing did not find any activations at the site of stimulation (De Weijer et al., 2014; Dowdle et al., 2018; Kemna and Gembris, 2003; Leitão et al., 2013; Xu et al., 2016), while only a single study that targeted areas outside M1 during rest and corrected for image artifacts using a field map reported local BOLD activations in a second-level analysis (Hawco et al., 2018).

Another critical issue to consider is the improvement in TMS technology over the years. Early studies using Dantec technology (Bohning et al., 2000b, 2000a, 1999, 1997) induced substantial dropout that likely substantially affected the SNR in the vicinity of the coil (Weiskopf et al., 2009). However, modern Magstim and especially MagVenture setups typically result in excellent signal underneath the TMS coil (Bergmann et al., 2021; De Weijer et al., 2014). Indeed, several studies have explicitly measured signal dropout using MagVenture equipment and have found it to be minimal (Leitão et al., 2013; Moisa et al., 2010, 2009). Further, the image distortions and dropout reduce with the distance from the coil. Given the typical scalp-to-cortex distance of 1-2 cm, the distortions in the brain are typically small (Bestmann et al., 2008b). Importantly, the signal drop even in early studies was not large enough to prevent the reliable detection of BOLD activations in M1 when that region was targeted (Bohning et al., 2000a, 2000b, 1999, 1998, 1997; De Weijer et al., 2014; Moisa et al., 2009). Thus, the image artifacts caused by the presence of the TMS coil appear unlikely to fully explain the lack of local BOLD increases uncovered in the present review, especially for studies that correct for image artifacts or use modern technology that has been shown to result in minimal levels of distortion.

Minor concerns

1) As a reader, it would be helpful if the authors provide a table describing Section 6 (i.e., animal studies). The table should include a similar format to other tables (e.g., species, TMS protocol, TMS location, findings, etc.).

We agree with the reviewers that adding a table in this section would be helpful and improve the manuscript. We have now added the following table to Section 6 of our manuscript:

Table 5. TMS studies performed in animals. The table lists all seven studies reporting TMS effects on neuronal activity. Five studies reported a combination of increasing and decreasing single neuron activity after stimulation. Two studies observed only increase in neuronal activity but the effects disappear after a few trials of single pulse stimulation. Arrows in the last column represent average increases and decreases in activity across the recorded neurons. FEF, frontal eye field; CFA, caudal forelimb area (rodent’s equivalent to the forelimb area of primate M1).

Study Species Target Protocol Anesthetized? N A combination of increased and decreased firing rate?

Moliadze et al. (2003) J Physiol Cat Primary visual cortex (area 17) Single pulse Yes 7 Yes ()

Allen et al. (2007) Science Cat Visual cortex TMS bursts (1-4 s, 1-8 Hz) Yes 8 No ()

Pasley et al. (2009) Neuron Cat Visual cortex TMS bursts (1-4 s, 1-8 Hz) Yes 2 No ()

Kozyrev et al. (2014) PNAS Cat Visual cortex Single pulse, rTMS (10 Hz) Yes 15 Yes ()

Mueller et al. (2014) Nat Neurosci Monkey FEF Single pulse No 2 Yes (neuron-dependent)

Li et al. (2017) Elife Rat CFA Single pulse Yes 17 Yes ()

Romero et al. (2019) Nat Commun Monkey Parietal cortex Single pulse No 2 Yes (neuron-dependent)

2) The authors should carefully correct the reference format (e.g., Allen RA, Psaley BN, Doung T, Freeman RD (2007) Transcranial magnetic stimulation elicits coupled neural and hemodynamic consequences. Science (1979) 317:1918-1921.).

The reference format is now corrected, and it is in accordance with the eNeuro reference style.

3) Line 90. Please mention how many studies have been added, i.e. published in 2020 and 2021.

According to our search, 5 new empirical articles were published in 2020 and 2021 (Cobos Sanchez et al., 2020; Jackson et al., 2021; Navarro de Lara et al., 2020; Rafiei et al., 2021; Scrivener et al., 2021), out of which only one study (Rafiei et al., 2021) provided information regarding BOLD signal changes in areas underneath the TMS coil. We have now added this information and are pasting the relevant sentence here (in response to the next comment we also paste the whole containing paragraph):

To do so, we started with the 69 studies reported in Bergmann et al. (2021) and then, using equivalent search criteria, looked for additional studies published in 2020 and 2021, which resulted in five additional studies (Cobos Sánchez et al., 2020; Jackson et al., 2021; Navarro de Lara et al., 2020; Rafiei et al., 2021; Scrivener et al., 2021).

4) Line 97. There seems to be a mismatch between the number of published TMS-fMRI and the studies discussed here. The authors state that they excluded 15 papers from the 69 studies published till 2019, i.e. discussed in by Bergmann and colleagues (Bergmann et al., 2021), plus the papers publishes in 2020 and 2021. However, the final included number of papers addressed in this review is 52. Can the authors explain this mismatch? Or even briefly discussed the findings of any paper that was not yet included in this review. Thank you!

We thank the reviewers for highlighting the mismatch. There are 69 studies in Bergman et al. review paper. We found five studies which were published in 2020 and 2021. This results in 74 studies in total. Out of these 74 studies, 5 studies were removed because they reported data from a single subject, and 13 studies were removed because they did not analyze BOLD signal changes under the coil. This results in 56 studies where two pairs of studies (Leitao et al., 2013, 2015 and Shitara et al., 2011, 2013) share a common dataset and in our manuscript, we only mention the ones (Leitao et al., 2015; Shitara et al., 2011) where they explicitly report the TMS effects underneath the coil. Therefore, we end up having 54 studies in our tables (not the 52 studies we had mentioned previously). Since our review is specifically focused on the issue of BOLD activation at the site of stimulation, we could not find a way to discuss the findings of the excluded studies without diverging too much from the main thread of the paper. In addition, we feel that such a discussion may induce too much overlap with the recent comprehensive review by Bergman et al. (2021). Because of that we have elected not to discuss in detail the excluded studies beyond citing them and pointing out the reason for not including them. Below we paste the relevant paragraph where all of these issues are mentioned:

Here we set to provide a comprehensive review of the published literature on concurrent TMS-fMRI with the goal of adjudicating between the two competing hypotheses above. First, we compiled all papers on concurrent TMS-fMRI published by December 31, 2021. To do so, we started with the 69 studies reported in Bergmann et al. (2021) and then, using equivalent search criteria, looked for additional studies published in 2020 and 2021, which resulted in five additional studies (Cobos Sánchez et al., 2020; Jackson et al., 2021; Navarro de Lara et al., 2020; Rafiei et al., 2021; Scrivener et al., 2021). Second, we excluded two sets of articles: (1) papers that primarily focused on methodological issues and only reported data from a single subject (Bestmann et al., 2006, 2003a; Navarro De Lara et al., 2015; Oh et al., 2019; Peters et al., 2013), and (2) papers that did not provide information that allows the determination of whether or not TMS led to an increase in local BOLD activation because relevant analyses were not reported (Chen et al., 2013; Cobos Sánchez et al., 2020; Eshel et al., 2020; Fonzo et al., 2017; Guller et al., 2012; Hanlon et al., 2016; Hermiller et al., 2020; Jackson et al., 2021; Navarro de Lara et al., 2020; Oathes et al., 2021; Ruff et al., 2008, 2006; Scrivener et al., 2021). Among the remaining studies, two pairs of studies shared the same underlying dataset (Leitão et al., 2013 and Leitão et al., 2015, as well as Shitara et al., 2011 and Shitara et al., 2013) and, therefore, we kept only the ones that explicitly mention results related to the presence of absence of activation at the site of stimulation (Leitão et al., 2015; Shitara et al., 2011). This process resulted in a total of 54 relevant articles. Of these, 22 articles delivered TMS outside of the primary motor (M1) or visual (V1) cortex at rest, 22 articles delivered TMS to M1 or V1 at rest, and 10 delivered TMS during a task.

5) Line 190: Can the authors motivate why “louder” pulses and “more pronounced tactile sensations” might elicit higher activation in the prefrontal cortex?

We thank the reviewers for bringing our attention to this sentence, which was indeed worded overly strongly by stating that higher intensity TMS pulses would necessarily be expected to produce higher PFC activity. We have now qualified this statement and also, as requested, expanded on the justification for why higher TMS intensities *may* induce non-specific increases in PFC. We also note that the argument here is not central to the paper (in our view, the main issue, which we highlight right after and elsewhere in the manuscript, is the lack of precise TMS localization in these papers), so if the reviewers don’t find our argument convincing, we would be happy to simply remove this statement from the manuscript. We paste below the updated section that includes the requested justification:

The first three papers targeted the prefrontal cortex and compared supra- to sub-threshold intensities using either first-level (Vink et al., 2018) or second-level analysis (Hawco et al., 2018; Li et al., 2004a). Each paper reported BOLD increases in many portions of the prefrontal cortex including near the presumed location of stimulation. However, higher intensity TMS is louder and elicits more pronounced tactile sensations compared to low intensity TMS. This makes high intensity TMS feel more uncomfortable, which may induce emotional or cognitive changes, with the prefrontal cortex known to be involved in both types of processes (Apkarian et al., 2005; Jobson et al., 2021; Lorenz et al., 2003; Miller et al., 2002).

6) Line 459: Does any of two studies discuss the net effect of the stimulation on the local activity?

The papers do include other measures beyond single neuron activity - including local field potentials, tissue oxygen, and total hemoglobin - which generally also showed increases. We now mention this specifically in the paper. We note, however, that the main issue that we highlight about these results is not whether there is a net effect but that the effects appear to diminish quickly with repeated stimulation. We paste the updated paragraph below:

Finally, two other studies delivered TMS to the visual cortex of anesthetized cats (Allen et al., 2007; Pasley et al., 2009). They found increased single neuron activity that lasted for a full minute (similar results were obtained using indirect measures of net activity including local field potentials, tissue oxygen, and total hemoglobin), but this effect diminished rapidly over the course of the experiment with some neurons showing no overall change in firing already by the fifth trial (Pasley et al., 2009). It should be noted that fMRI studies typically deliver hundreds of TMS trials, and therefore the BOLD effects in these studies primarily reflect the steady-state TMS influence on neural firing achieved after several dozen trials. Finally, both of these studies were conducted in anesthetized animals, leaving open the question of whether the same neuronal effects would be observed in the awake brain.

7) Line 474. Section 7. The authors should also discuss the fact that most of the TMS-fMRI studies tested a relatively low number of subjects, e.g. compared with the number of subjects employed in standard fMRI studies. For example, in the three studies published most recently, i.e. Jung et al. 2020 (12 subjects for each experimental group), Peters et al. 2020 (4 subjects), Rafiei et al. 2021 (5 subjects in Experiment 1 and 6 subjects in Experiment 2). It is true that this is not relevant for individual effects of the stimulation; however, this could be a potential issue when addressing group level effects of TMS at the stimulation site.

We agree with the reviewers that the low number of subjects in concurrent TMS-fMRI studies is a concern and that it should be discussed in Section 7 of our manuscript. We have now added a new subsection titled “Low number of subjects” and discussed this issue as one of the limitations and potential causes for the failure to find activation under the coil. Nevertheless, we highlight several reasons why this issue alone does not seem to explain the pattern of results in the literature. We paste the new subsection below:

Low number of subjects

A fourth possibility for the lack of BOLD increase at the site of stimulation is that most concurrent TMS-fMRI studies employ a low number of subjects, and this results in low statistical power. Indeed, the median sample size among the 52 studies reviewed here is 8. Importantly, this concern does not apply to the studies that examined the effects of TMS on each individual, but they do affect the studies that performed group-level analyses. Nevertheless, it should be noted that M1 activation is robust even in studies with few number of subjects (Bohning et al., 2003, 2000a, 1998; De Weijer et al., 2014; Moisa et al., 2009) whereas even the top three studies with largest number of subjects (Jung et al., 2016; Kearney-Ramos et al., 2018; Mason et al., 2014) did not find significant activation when TMS was applied over areas outside of M1/V1. Thus, while the low number of subjects remains an important concern, this issue alone does not seem to explain the pattern of results in the literature.
